# N-terminal pro-B-type natriuretic peptide levels vary by ethnicity and are associated with insulin sensitivity after gestational diabetes mellitus

**DOI:** 10.1186/s12933-024-02349-1

**Published:** 2024-08-03

**Authors:** Archana Sharma, Kåre I. Birkeland, Ingrid Nermoen, Christine Sommer, Elisabeth Qvigstad, Sindre Lee-Ødegård, Kari A. Sveen, Naveed Sattar, Stina T. Sollid, Torbjørn Omland, Peder L. Myhre

**Affiliations:** 1 Department of Endocrinology, Akershus University Hospital, Lørenskog, Norway; 2 Institute of Clinical Medicine, University of Oslo, Oslo, Norway; 3 Department of Endocrinology, Morbid Obesity and Preventive Medicine, Oslo University Hospital, Oslo, Norway; 4 Institute of Cardiovascular and Medical Sciences, BHF Glasgow Cardiovascular Research Centre, University of Glasgow, 126 University Place, Glasgow, G12 8TA UK; 5 Department of Medicine, Drammen Hospital, Vestre Viken Health Trust, Drammen, Norway; 6 Department of Cardiology, Akershus University Hospital, Lørenskog, Norway; 7 Department of Endocrinology, Campus Akershus University Hospital, Lørenskog, 1478 Norway

**Keywords:** NT-proBNP, Ethnicity, Gestational diabetes mellitus, Heart disease, Insulin sensitivity, Normoglycemia, Prediabetes, Prevention

## Abstract

**Background:**

Individuals of South Asian origin have a greater risk of cardiovascular disease after gestational diabetes mellitus (GDM) than European individuals. B-type natriuretic peptide (BNP) and the amino-terminal fragment of its prohormone (NT-proBNP) are commonly used for heart failure screening and diagnosis, but biologically BNP exerts several beneficial cardiovascular effects primarily by counteracting the renin-angiotensin-aldosterone-system. We asked whether ethnic differences in circulating NT-proBNP levels could be explained by the differences in cardiometabolic and inflammatory risk markers?

**Methods:**

We examined 162 South Asian and 107 Nordic women in Norway 1–3 years after GDM with a clinical examination, fasting blood samples and an oral glucose tolerance test. We measured the levels of NT-proBNP, high-sensitivity cardiac troponin T, high-sensitivity C-reactive protein (hsCRP), interleukin-6 (IL-6), leptin, adiponectin and markers of insulin sensitivity, such as the Matsuda insulin sensitivity index (ISI). Finally, we tried to identify which independent covariate best mediated the ethnic differences in NT-proBNP.

**Results:**

The mean (SD) age was 35.3 (4.5) years, BMI 29.1 (6.0) kg/m^2^, waist-height ratio 0.60 (0.08) and 164 women (61%) had prediabetes/diabetes. Notably, South Asian women had lower levels of NT-proBNP than Nordic women in both the normoglycemic and prediabetes/diabetes groups (median (IQR) 26  (15–38)  vs. 42 (22–66) ng/L, *p* < 0.001). Higher NT-proBNP levels were associated with greater insulin sensitivity in both South Asian and Nordic women (*p* = 0.005 and *p* < 0.001). South Asian women had higher levels of hsCRP (median (IQR) 2.2 (1.1–4.4) vs. 1.2 (0.3–4.2) mg/L), IL-6 (2.3 (1.5–3.2) vs. 1.5 (1.5–2.5) pg/mL), leptin (1647 (1176–2480) vs. 1223 (876–2313) pmol/L), and lower adiponectin levels (7.2 (5.3–9.3) vs. 10.0 (7.2–13.5) mg/L) and Matsuda ISI (2.4 (1.7–3.7) vs. 4.2 (2.9–6.1), p_all_<0.01) than Nordic women. Even after adjusting for these differences, higher NT-proBNP levels remained associated with insulin sensitivity (22% higher NT-proBNP per SD Matsuda ISI, *p* = 0.015). Insulin sensitivity and adiponectin mediated 53% and 41% of the ethnic difference in NT-proBNP.

**Conclusions:**

NT-proBNP levels are lower in South Asian than in Nordic women after GDM. Lower NT-proBNP levels correlate with impaired insulin sensitivity. Lower NT-proBNP levels in South Asian women could, therefore, be attributed to impaired insulin sensitivity rather than total body fat.

**Supplementary Information:**

The online version contains supplementary material available at 10.1186/s12933-024-02349-1.

## Background

Individuals of South Asian origin in high-income countries have greater risk of cardiovascular disease than individuals of European descent [1]. Gestational diabetes mellitus (GDM) is more prevalent in South Asian women [2, 3], and is a well-described risk factor for future ischemic heart disease and heart failure [4–6].

Natriuretic peptides (NPs) exert several beneficial effects on the cardiovascular system, including vasodilation, diuresis, natriuresis, and suppression of the renin–angiotensin–aldosterone-system (RAAS) [7]. Accordingly, NP levels below or within the lower physiological range are also associated with a worse cardiometabolic risk profile in individuals without apparent cardiovascular disease [7, 8]. The biologically active B-type NP (BNP) and the inactive N-terminal fragment of the prohormone of BNP (NT-proBNP) are released into the bloodstream at equimolar concentrations [8]. NT-proBNP play an important role in diagnosing heart failure and assessing future cardiovascular risk. Higher levels of NT-proBNP associate with an increased risk of incident heart failure and worse outcomes [9–11]. Recent guidelines recommend screening individuals at risk of heart failure with NT-proBNP, including patients with diabetes [11, 12].

Interestingly, prospective cohort studies have reported that NT-proBNP levels are lower in individuals with obesity or diabetes, suggesting that obesity and low insulin sensitivity may have negative effects on the neurohormonal homeostasis provided by the NP [8, 13, 14]. Mendelian randomization studies also reveal a causal link between low NP levels and the development of type 2 diabetes [15, 16]. Furthermore, NPs reportedly increase hepatic and muscle lipid oxidation, and NP deficiency may, therefore, increase lipid-induced reduction of insulin sensitivity [17]. The latter is believed to be an important factor in the development of diabetes in South Asian individuals [18]. Impaired insulin sensitivity, in general, is linked to ectopic fat and adipocyte hypertrophy, particularly in South Asian individuals [18]. Impaired insulin sensitivity is also associated with chronic inflammation, which is more common in South Asian populations [19]. Moreover, cardiac NPs have been shown to reduce adipose tissue inflammation by modifying adipokine secretion and, thereby improving insulin sensitivity [7].

Whether the known ethnic differences in cardiometabolic and inflammatory risk markers can account for potential differences in NT-proBNP levels have not been evaluated, although such analysis may provide valuable insights into cardiovascular risk assessments and treatment decisions.

We, therefore, aimed to compare ethnic differences in the serum concentrations of NT-proBNP and assess the role of cardiometabolic and inflammatory risk markers in explaining the potential differences between South Asian and Nordic women with normal glucose tolerance and prediabetes or diabetes 1–3 years after a GDM pregnancy.

## Methods

The *DIA*betes in *S*outh *A*sians 1 (DIASA 1) trial was approved by the South-Eastern Norway Regional Committee for Medical and Health Research Ethics (reference number: 2018/689). All participants provided written informed consent.

### Design, study population and data collection

As described in detail previously [20], between September 1, 2018, and December 31, 2021, we invited South Asian (Pakistan, India, Bangladesh, or Sri Lanka) and Nordic (Norway, Sweden, Denmark, Finland, or Iceland) women with a diagnosis of GDM in their last pregnancy (according to the WHO 1999 [14] or modified International Association of Diabetes and Pregnancy Study Group (IADPSG) criteria [15]) who delivered 12–36 (± 3) months previously at one of three hospitals in the Oslo area, Norway. All participants were interviewed and underwent a physical examination at baseline. Medical history and supplementary information were retrieved from the electronic medical records.

Height, weight, waist and hip circumferences [16] were measured at the study visit.

### Laboratory analyses

All women underwent an oral glucose tolerance test (OGTT) after at least eight hours of fasting. Blood was collected in cooled sodium fluoride tubes for glucose analysis and kept on ice until centrifugation at 4℃; and in serum-separating tubes for analyses of other biomarkers and centrifuged after 30 min. Plasma glucose was analysed by enzymatic photometry (Roche Diagnostics, Mannheim, Germany), and serum C-peptide and insulin were analysed by electrochemiluminescence immunoassay (cobas e601, Roche Diagnostics). The coefficients of variation (CVs) were 2.5%, 7%, and 4–5% for glucose, insulin, and C-peptide, respectively. Serum total cholesterol, high-density lipoprotein-cholesterol (HDL-C), low-density lipoprotein-cholesterol (LDL-C), and total triglycerides were analysed by enzymatic photometry (Roche Diagnostics). The CVs were 3%, 4%, 3.5% and 4%, respectively. Serum leptin levels were measured by an enzyme-linked immunosorbent assay (ELISA) kit (Mediagnost) with a CV of 10%. Serum adiponectin levels were analysed by a competitive radioimmunoassay kit (Merck Millipore) with a CV of 6.6%. Serum blood samples of high-sensitivity (hs) cTnT, NT-proBNP, interleukin 6 (IL-6), and hs-C-reactive protein (hsCRP) were analysed using electrochemiluminescence immunoassays on the cobas e 801 platform (Roche Diagnostics). Hs-cTnT was measured by the Elecsys STAT immunoassay with a CV of 3.9% at 11.8 ng/L and 3.0% at 89 ng/L. The value of 1.5 ng/L was imputed for women with undetectable hs-cTnT values defined by the manufacture as values below 3 ng/L. NT-proBNP was measured by the Elecsys proBNP II STAT immunoassay [21], and the CV reported by the manufacturer was 2.5% at 127 ng/L and 1.3% at 1706 ng/L. The value of 2.5 ng/L was imputed for women with undetectable NT-proBNP values defined by the manufacture as values below 5 ng/L. IL-6 was measured by the Elecsys IL-6 immunoassay with a CV of 4.9% at 6.4 pg/mL and 1.4% at 189 pg/mL. HsCRP was measured by the high-sensitivity Elecsys assay with a CV reported as 3.3% < 5 mg/L and 1-5.3% ≥ 5 mg/L.

All laboratory analyses were performed at Oslo University Hospital, Aker, except for hs-cTnT, NT-proBNP, IL-6 and hsCRP, which were performed at Akershus University Hospital.

### Definitions and calculations

We used the following definitions for body mass index (BMI) categories: normal weight (BMI < 23 kg/m^2^ for South Asian women, BMI < 25 kg/m^2^ for Nordic women), overweight/obesity (BMI ≥ 23 kg/m^2^ for South Asian women, BMI ≥ 25 kg/m^2^ for Nordic women [22, 23]).

Prediabetes was defined according to the WHO-International Expert Committee criteria: fasting plasma glucose (FPG) 6.1–6.9 mmol/L and/or 2-h plasma glucose 7.8–11.0 mmol/L and/or HbA_1c_ 42–47 mmol/mol (6.0-6.4%) [14, 17]. Diabetes status was defined according to international standards but based on a single measurement of elevated glucose [18, 19].

Participants with prediabetes or diabetes were clustered together in the DIASA1 study due to the low number of women with diabetes [20].

Insulin sensitivity was estimated using the homeostatic model assessment2-sensitivity (HOMA2-S) [24, 25] and by calculating the Matsuda insulin sensitivity index (Matsuda ISI) as 10,000/√ (fasting serum insulin [uIU/mL] × FPG [mg/dL]) × (mean OGTT insulin [uIU/mL]) × (mean OGTT glucose [mg/dL]) [24].

We defined low insulin sensitivity as Matsuda ISI ≤ 1.9 (calculated as values ≤ 25% quartile).

### Statistical analyses

Characteristics were presented as mean (SD), median (interquartile range (25-75th percentile), IQR), or number [%]. To compare differences between groups, we used independent t-tests for normally distributed data, Mann-Whitney tests for non-normally distributed data, and Fisher’s exact tests for categorical variables. Variables were log-transformed to approximate normality if necessary. We used boxplots of NT-proBNP levels to visualize ethnic differences by (1) BMI categories, (2) insulin sensitivity and (3) glucose tolerance. Linear regression analyses were performed to identify determinants of NT-proBNP levels. Covariates with p values ≤ 0.25 or theoretically relevant covariates were included in multivariable regression analyses. Model 1 was adjusted for known confounders for NT-proBNP: ethnicity, age, systolic blood pressure, and estimated glomerular filtration rate (eGFR). Model 2 was adjusted for variables in Model 1 + IL-6, and not hsCRP due to a high correlation between the two. As leptin correlates closely with body fat, and BMI reflects many other dimensions of the body weight such as lean mass and is difficult to compare across ethnicities, we used only leptin (and not BMI) in the regression analyses [22, 26]. Therefore, Model 3 was adjusted for variables in Model 2 + leptin levels. Model 4 was adjusted for variables in Model 3 + adiponectin levels. Finally, Model 5 was adjusted for variables in Model 4 + insulin sensitivity (by Matsuda ISI). We performed separate analyses of South Asian and Nordic women, and of normoglycemic and prediabetes/diabetes women. The results were expressed as beta-coefficients with 95% CI. Because we used log-transformed dependent variables, we exponentiated the beta-coefficient for the independent variables to characterise its multiplicative effect on the absolute NT-proBNP levels.

Finally, we performed a mediation analysis to provide information about how much of the exposure difference (i.e., ethnic difference) to an outcome (i.e., NT-proBNP) is due to a covariate (e.g., insulin sensitivity). The mediator is a covariate that is affected by the exposure and affects the outcome. If the relationship between the exposure and the outcome decreases or becomes nonsignificant, this relationship is partially or completely mediated through the mediator. Several parallel mediators were examined: age, BMI, systolic blood pressure, eGFR, IL-6, hsCRP, leptin, adiponectin, and insulin sensitivity (by Matsuda ISI).

For all analyses p-values < 0.05 were considered statistically significant, but for the correlation and interaction analyses a p-value < 0.001 was used to reduce the number of sporadic findings as correlations and interactions among all the covariates in Model 5 were examined. SPSS 27 and STATA 17 were used for the statistical analyses.

## Results

### Baseline characteristics

At a median (IQR) of 16.8 (13.4–25.6) months after GDM 70% of the South Asian and 47% of the Nordic women had prediabetes or diabetes (*p* < 0.001). BMI did not differ between the ethnic groups, but the proportion of overweight and obesity by ethnic-specific BMI criteria was higher in South Asian than in Nordic women. South Asian women were younger and had higher waist-to-height ratio than comparable Nordic women. The South Asian women also had lower systolic blood pressure and HDL cholesterol levels, and higher HbA1c, insulin, triglycerides and eGFR levels than the Nordic participants (Table 1).

**Table 1:** The participants’ characteristics by ethnicity

	South Asian *n* = 162 [60%]	Nordic *n* = 107 [40%]	*p* value
Age (years)	34.6 (4.1)	36.5 (4.9)	< 0.001
Time since index pregnancy (months)^‡^	16.1 (13.1–25.4)	18.8 (14.8–25.9)	0.074
Weight (kg)	73.9 (14.6)	81.7 (19.7)	< 0.001
Height (cm)	159.5 (6.4)	166.9 (6.1)	< 0.001
BMI (kg/m^2^)	29.0 (5.3)	29.3 (6.9)	0.676
Ethnic-specific overweight/obesity	150 [93]	75 [70]	< 0.001
Waist-to-height ratio	0.61 (0.07)	0.58 (0.09)	0.002
Systolic blood pressure (mmHg)	115 (10)	118 (11)	0.015
Diastolic blood pressure^‡^ (mmHg)	75 (70–80)	77 (72–82)	0.080
Fasting plasma glucose (mmol/L)	5.8 (0.7)	5.7 (0.8)	0.150
2-h OGTT glucose (mmol/L)	8.7 (2.7)	7.9 (2.6)	0.013
HbA_1c_ (mmol/mol)	39 (5)	36 (4)	< 0.001
HbA_1c_ [%]	[5.7 (2.6)]	[5.4 (2.5)]	< 0.001
Prediabetes/diabetes	114 [70]	50 [47]	< 0.001
Fasting insulin^‡^ (pmol/L)	104 (74–154)	64 (45–99)	< 0.001
Fasting c-peptide (pmol/L)	1006 (304)	836 (330)	< 0.001
Total cholesterol (mmol/L)	4.4 (0.7)	4.3 (0.8)	0.374
Triglycerides^‡^ (mmol/L)	1.10 (0.83–1.60)	0.90 (0.70–1.20)	< 0.001
High density lipoprotein cholesterol (mmol/L)	1.13 (0.26)	1.28 (0.31)	< 0.001
Low density lipoprotein cholesterol (mmol/L)	2.97 (0.65)	2.89 (0.78)	0.353
eGFR (mL/min/1.73m^2^)	117 (10)	111 (11)	< 0.001

Patient characteristics are presented as mean and (standard deviation, SD) or ^‡^median and (25-75th percentile) or number (n) and [%]

Ethnicity-specific overweight/obesity: BMI ≥ 23 kg/m^2^ for South Asian women, BMI ≥ 25 kg/m^2^ for Nordic women

### Ethnic differences in NT-proBNP

The median (IQR) NT-proBNP level in the total population was 31 (17–50) ng/L. South Asian women had lower levels of NT-proBNP than Nordic women in both the normoglycemic (median (IQR) 29 [17–37] vs. 40 (22–58) ng/L, *p* = 0.005) and prediabetes/diabetes groups (22 [14–39] vs. 44 (21–74) ng/L, *p* < 0.001). Three (2%) South Asian and six (6%) Nordic women had NT-proBNP levels ≥ 125 ng/L, which is a cut-off used to signify high risk of heart failure. A total of 15 (9 South Asian and 6 Nordic) women had NT-proBNP levels < 5 ng/L. Hs-cTnT concentrations were below the lower detection level in > 95% of participants, and we found no difference in hs-cTnT levels between South Asian and Nordic women (median (IQR) < 3 (< 3-<3) ng/L vs. < 3 (< 3-<3) ng/L, *p* = 0.709) (Table 2, Additional file 1: Table S1 and Fig. S1).

**Table 2:** Ethnic differences in cardiac, inflammation, adipokine and insulin sensitivity markers

			South Asian	Nordic	*p*-value
			*n* = 162	*n* = 107	
Cardiac		NT-proBNP (ng/L)	26 (15–38)	42 (22–66)	< 0.001
		Troponin T (ng/L)	< 3 (< 3-<3)	< 3 (< 3-<3)	0.709
Inflammation		CRP (mg/L)	2.2 (1.1–4.4)	1.2 (0.3–4.2)	0.003
		Interleukin 6 (pg/mL)	2.3 (1.5–3.2)	1.5 (1.5–2.5)	< 0.001
Adipokines		Leptin (pmol/L)	1647 (1176–2480)	1223 (876–2313)	0.004
		Adiponectin (mg/L)	7.2 (5.3–9.3)	10.0 (7.2–13.5)	< 0.001
Insulin sensitivity		HOMA2-S	50 (35–74)	82 (54–116)	< 0.001
		Matsuda-ISI	2.4 (1.7–3.7)	4.2 (2.9–6.1)	< 0.001

Data presented as medians (25-75th percentiles) or numbers (n) and [%]

HOMA2-S: HOMA2-sensitivity, ISI: insulin sensitivity index, NT-pro BNP: N-terminal pro B-type natriuretic peptide. *p*-value for South Asian vs. Nordic women

South Asian women had significantly higher levels of hsCRP, IL-6 and leptin, and significantly lower adiponectin levels than Nordic women (Table 2). Normoglycemic South Asian women had similar concentrations of hsCRP, IL-6, leptin and adiponectin as women with prediabetes or diabetes (Supplementary Table 1). All markers of insulin sensitivity were significantly lower in South Asian than in Nordic women (Table 2), independent of glucose tolerance status (Additional file 1: Table S1).

### Relationship with ethnic-specific BMI and insulin sensitivity

**Fig. 1:**
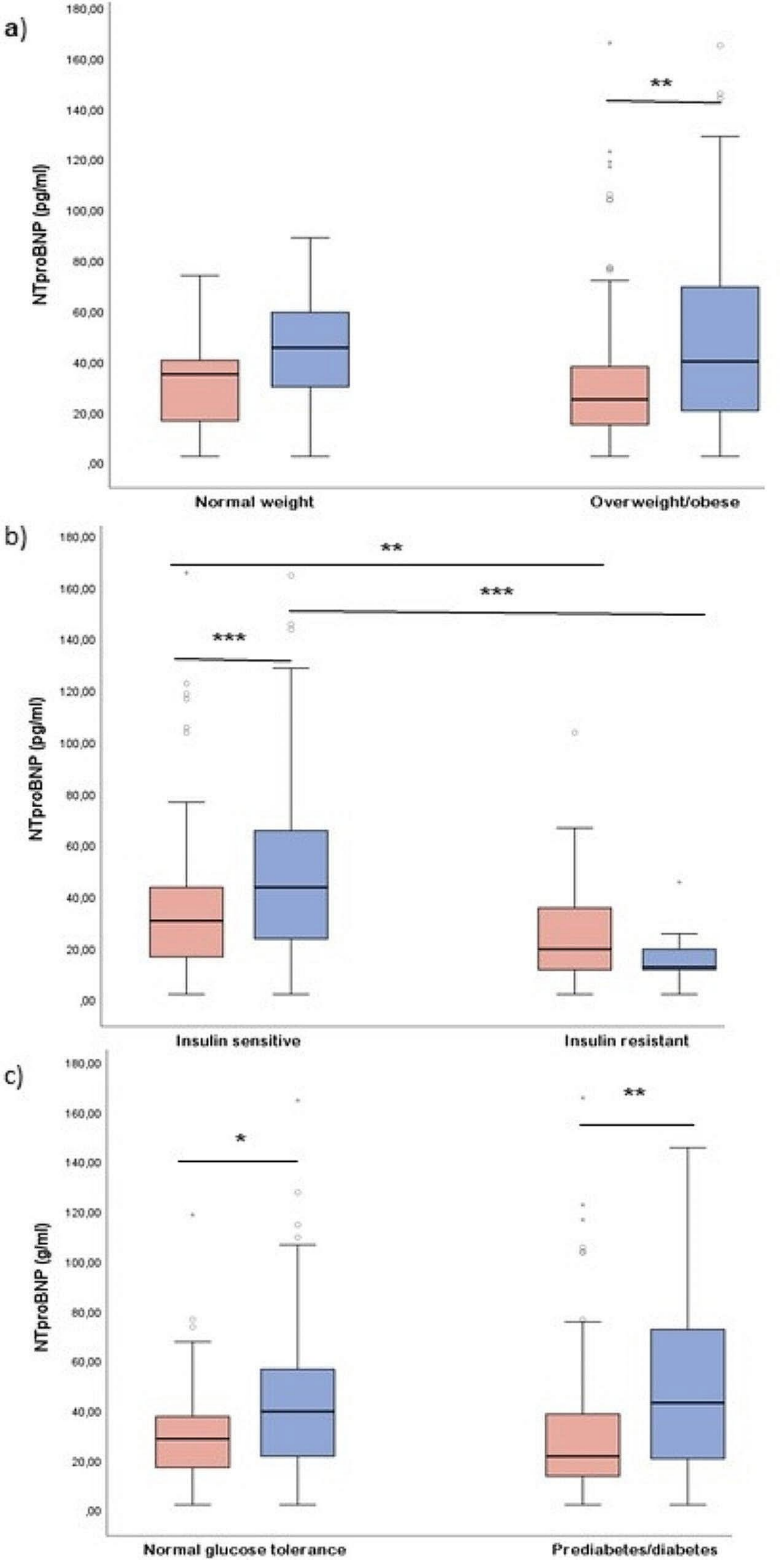
Median NT-proBNP levels (ng/L) by (A) ethnicity-specific BMI categories (overweight/obesity: BMI ≥ 23 kg/m^2^ for South Asian women, BMI ≥ 25 kg/m^2^ for Nordic women) (B) insulin sensitivity Index (insulin-resistant: Matsuda ISI ≤ 1.9) and (C) glucose tolerance (prediabetes/diabetes: FPG > 6.1 mmol/L and/or 2-h plasma glucose ≥ 7.8 mmol/L and/or HbA_1c_ ≥ 42 mmol/mol (≥ 6.0%)) in South Asian (red) and Nordic women (blue) after gestational diabetes. *p-value < 0.05, **p-value < 0.01, *** p-value < 0.001

In the total population, NT-proBNP levels did not vary across BMI categories (Fig. 1).

In women who were overweight or obese, South Asian women had lower NT-proBNP levels than Nordic women (Fig. 1A). In women with normal insulin sensitivity or normal glucose tolerance, South Asian had lower NT-proBNP levels than Nordic women (Fig. 1B and C). Among women with impaired insulin sensitivity by Matsuda ISI, we found no differences in NT-proBNP levels between the ethnicities (Fig. 1B), but in women with prediabetes or diabetes South Asian women had lower NT-proBNP levels than Nordic women (Fig. 1C).

NT-proBNP levels were higher in insulin-sensitive than in insulin-resistant individuals among both South Asian and Nordic women (Fig. 1B).

### Predictors of NT-proBNP

NT-proBNP correlated with eGFR (*r*=-0.206, *p* < 0.001), fasting s-insulin (*r*=-0.361, *p* < 0.001), adiponectin (*r* = 0.293, *p* < 0.001), HOMA2-S (*r* = 0.355, *p* < 0.001), and Matsuda-ISI (*r* = 0.369, *p* < 0.001), but not significantly with BMI (*r*=-0.065, *p* = 0.287) (Additional file 1: Table S2).

As shown in Table 3, after adjusting for known confounders for NT-proBNP (Model 1), inflammatory markers (Model 2) and leptin levels (Model 3), Nordic ethnicity remained associated with higher NT-proBNP levels (*p* = 0.027). This association was lost when we further adjusted for adiponectin levels (Model 4) and insulin sensitivity (Model 5). Then, only higher Matsuda ISI (22% higher NT-proBNP per SD Matsuda ISI, *p* = 0.015) and higher systolic blood pressure (15% higher NT-proBNP levels per SD higher systolic blood pressure, *p* = 0.039) were associated with higher NT-proBNP levels (Table 3). In relation to NT-proBNP, we found a significant interaction between leptin levels and insulin sensitivity (Matsuda ISI) (p_interaction_ <0.001), such that NT-proBNP levels did not increase with higher leptin levels in women with low insulin sensitivity.

**Table 3:** Predictors of NT-proBNP (ng/L) concentrations (log transformed) in unadjusted and adjusted linear regression analyses

	Unadjusted		Model 1		Model 2		Model 3		Model 4		Model 5	
	Crude B	*p*	Std B	*p*	Std B	*p*	Std B	*p*	Std B	*p*	Std B	*P*
Ethnicity	−0.201	< 0.001	−0.155	0.015	−0.161	0.011	−0.148	0.027	−0.096	0.171	−0.031	0.686
Age (years)	0.116	0.057	0.032	0.632	0.041	0.532	0.029	0.673	0.023	0.740	0.006	0.927
Systolic blood pressure (mmHg)	0.105	0.085	0.065	0.286	0.093	0.124	0.141	0.027	0.120	0.061	0.139	0.039
eGFR (mL/min/1.73m^2^)	−0.171	0.005	** −**0.117	0.084	−0.116	0.085	−0.136	0.053	−0.116	0.102	-0.114	0.115
Interleukin-6 (pg/mL)	−0.064	0.294			−0.022	0.711	0.051	0.437	0.031	0.633	0.040	0.547
Leptin (pmol/L)	−0.155	0.015					−0.178	0.009	−0.131	0.052	-0.067	0.358
Adiponectin (mg/L)	0.238	< 0.001							0.162	0.018	0.109	0.132
Matsuda ISI	0.265	< 0.001									0.200	0.015

Model 1 (ethnicity, age, systolic blood pressure, eGFR), model 2 (model 1 + interleukin − 6), model 3 (model 2 + leptin), model 4 (model 3 + adiponectin) and model 5 (model 4 + Matsuda ISI (insulin sensitivity index))

For all analyses p-values < 0.05 were considered significant

A sensitivity analysis replacing leptin with BMI was performed to link the more common used BMI to NT-proBNP levels. This did not alter our results substantially (Additional file 1: Table S3).

In the subgroup analysis of South Asian women, higher Matsuda ISI (24% higher NT-proBNP levels per SD higher Matsuda ISI (*p* = 0.030)), lower leptin levels (23% lower NT-proBNP levels per SD higher leptin (*p* = 0.009)) and higher IL-6 levels (20% higher NT-proBNP levels per SD higher IL-6 levels (*p* = 0.047)) were associated with higher NT-proBNP levels in the fully adjusted Model 5 (Additional file 1: Table S4).

In Nordic women, only higher Matsuda ISI (32% higher NT-proBNP levels per SD higher Matsuda ISI (*p* = 0.020) was associated with higher NT-proBNP levels in the fully adjusted Model 5 (Additional file 1: Table S4).

In women with normal glucose tolerance, lower leptin levels (24% lower NT-proBNP levels per SD higher leptin (*p* = 0.030)) and lower age (22% lower NT-proBNP levels per SD higher age (*p* = 0.038)) were associated with higher NT-proBNP levels in the fully adjusted Model 5 (Additional file 1: Table S5). In the prediabetes and diabetes group, greater Matsuda ISI (38% higher NT-proBNP levels per SD higher Matsuda ISI (*p* < 0.001)) turned out to be the most important factor for predicting higher NT-proBNP levels after adjusting for all variables (Additional file 1: Table S5).

### Mediation analyses of ethnic differences in NT-proBNP

We tested whether some of the associated phenotypic traits or biomarkers could mediate the ethnic differences shown in NT-proBNP. We found that insulin sensitivity (by Matsuda ISI), adiponectin and eGFR mediated 53%, 41% and 14%, respectively, of the ethnic difference in NT-proBNP levels (Additional file 1: Table S6).

## Discussion

In this cohort of women 1–3 years after GDM, we demonstrate that South Asian women have lower NT-proBNP levels compared to Nordic women. This was related to South Asian women’s lower insulin sensitivity and unfavourable adipokine profile, as the majority of the difference in NT-proBNP levels could be explained by these factors. These findings may be important when evaluating NT-proBNP levels in South Asian and insulin resistant individuals, and enhance our understanding of cardiovascular disease in individuals of South Asian origin.

### Ethnic differences in NT-proBNP

South Asian women had lower NT-proBNP levels than Nordic women. This finding was consistent in both women with normoglycemia and prediabetes/diabetes and is in line with previous data on ethnic differences in NT-proBNP levels [27, 28]. These studies suggest that differences in NT-proBNP processing and clearance are possible mechanisms behind the lower levels in young healthy African vs. White American individuals. We add novel data to this field by demonstrating that the difference in ethnicity was related to South Asian women’s impaired insulin sensitivity and an unfavourable adipokine profile. We are not aware of previous studies that have reported similar findings.

Although a positive association between NT-proBNP levels and insulin sensitivity could be expected [29–32], the link to ethnic differences has not been previously described. Importantly, regular measurements of NT-proBNP to assess heart failure risk in patients with diabetes have recently been recommended by the American Diabetes Association (ADA) [11, 12], and concentrations ≥ 125 ng/L have been suggested as a threshold to identify individuals at risk, independent of glucose levels [33, 34]. Our findings highlight the need for studies to establish the impact of ethnicity on cut-off levels for NT-proBNP to assess cardiovascular risk, which also is in accordance with available literature [27, 28].

Impaired insulin sensitivity [18] and an unfavourable inflammatory [19, 35] and adipokine profile [36] have been demonstrated to be present years before the diagnosis of prediabetes or type 2 diabetes among South Asian individuals. Accordingly, we found lower hepatic and whole-insulin sensitivity, and adverse inflammatory and adipokine profiles at a younger age in South Asian vs. Nordic normoglycemic women. This may explain the younger age of onset of heart failure in South Asian than in Western individuals [37] as data show that higher leptin and lower adiponectin levels are associated with activation of the RAAS system and volume overload [38]. Furthermore, the well-known lower insulin sensitivity linked to factors such as more ectopic fat [18, 39, 40] and lower muscle mass [41] in South Asian individuals may reflect their lower NT-proBNP levels as NPs are suggested to enhance lipolysis and energy expenditure [7, 8].

### Predictors of NT-proBNP levels

The difference in NT-proBNP levels among ethnicities did not persist after adjustment for the lower insulin sensitivity and unfavourable adipokine profile in South Asian women. In accordance with previous studies [30, 31] and with our correlation analyses, although a low correlation was found, impaired insulin sensitivity was the factor that most strongly associated with NT-proBNP levels [42]. As 30% of symptomatic heart failure patients with preserved ejection fraction have normal NT-proBNP levels (< 100 ng/L) [43], it would be of interest to investigate whether low insulin sensitivity could explain a part of this paradox.

We did not find an association between levels of inflammatory markers and NT-proBNP. There was a correlation between lower IL-6 and higher NT-proBNP levels, but this did not persist in adjusted models. In patients with heart failure, however, higher concentration of inflammatory markers associate with worse outcomes and higher levels of natriuretic peptides [44]. As our cohort of women was substantially younger, without heart failure and low NT-proBNP levels, we believe the lack of association reflects the differential information provided by natriuretic peptides depending on the clinical setting.

The interaction analysis showed that higher leptin levels were associated with lower NT-proBNP levels in individuals with low insulin sensitivity. Higher leptin levels correlate well with higher body fat [26]. Furthermore, obese individuals have increased levels of circulating and membrane-bound NP receptor C (NPR-C) in adipocytes [45], which aids in NP clearance and could explain our finding of lower NT-proBNP levels with higher leptin levels in South Asian women. However, in contrast to BNP, NT-proBNP is not cleared by NPR-C. The clearance mechanisms of NT-proBNP is unclear, but it is postulated that > 50% of the total clearance occurs in renal tissue [46]. In our multivariate analyses, we adjusted for ethnic differences in eGFR. We did not measure other clearance mechanisms for NT-proBNP. Therefore, further research on this topic is needed. We also found that higher adiponectin levels were associated with higher NT-proBNP levels.

Our findings are in line with previous data [7] and indicate that cardiac NP activation may modulate adipokine secretion and low-grade inflammation in adipose tissue leading to improved insulin sensitivity. This theory is further supported by our data as higher leptin (or body fat) levels were important for predicting low NT-proBNP levels in women with normal glucose tolerance, while impaired insulin sensitivity was the most important factor after developing prediabetes/diabetes.

### Mediation analyses of ethnic differences in NT-proBNP

Adjustment for ethnic differences in body fat (leptin levels) attenuated the ethnic difference in NT-proBNP levels. However, adjustment for insulin sensitivity and adiponectin levels were the only factors that made this difference nonsignificant. These findings again suggest that impaired insulin sensitivity was the most important factor explaining South Asian women’s lower NT-proBNP levels, followed by an adverse adiponectin profile and a higher renal filtration rate compared to Nordic women. Our findings thus lend support to initiatives that recommend strong preventive measures against central fat accumulation and thereby impaired insulin sensitivity, as central and ectopic fat are thought to be instrumental for increased diabetes risk in South Asian populations. Importantly, lifestyle intervention in the Diabetes Prevention Program (DPP) study was associated higher cardiac NT-proBNP levels with improved insulin sensitivity, independent of weight status [30]. Impaired insulin sensitivity and glycemic control are further linked to increased heart failure risk, even in individuals with recent-onset diabetes or at a younger age [11], and may, therefore, account for a substantial fraction of the disparities in cardiovascular disease incidence between South Asian and White populations [1, 47].

### Limitations

A limitation of our study is the cross-sectional design, and we cannot exclude confounding or reverse causation. Second, we did not measure other natriuretic peptides, such as ANP or BNP due to shorter half-life. However, previous reports suggest high correlation between NT-proBNP and the other cardiac natriuretic peptides [27, 29]. Third, no data on cardiac structure or function were available. Some women, particularly those with obesity, may have subclinical diastolic dysfunction, although this is less likely due to their young age and overall low NT-pro-BNP levels. Fourth, we lack data on potential confounders like dietary salt intake and levels of physical activity. Further, we defined glucose tolerance by the WHO criteria, in order to have one definition for the whole cohort. We believe this is the most correct approach for two reasons: [1] The WHO criteria are recommended in the Nordic population; and [2] previous studies have demonstrated that the excess cardiovascular risk in individuals with prediabetes is mainly explained by other cardiometabolic risk factors beyond hyperglycemia [48]. Some of our findings may, therefore, not applicable to women with prediabetes defined by the ADA criteria. Finally, no information on genetic factors that might influence NT-proBNP levels was available, such as differences in the NPPB (promoter region of the BNP) gene allele.

## Conclusions

South Asian women had lower NT-proBNP levels than Nordic women after GDM. This difference was related to South Asian women’s lower insulin sensitivity and unfavourable adipokine profile. These findings suggest that ethnicity and insulin sensitivity should be taken into account when interpreting NT-proBNP levels.

### Electronic supplementary material

Supplementary Material 1

Supplementary Material 2

## Data Availability

No datasets were generated or analysed during the current study.

## Abbreviations

CV: Coefficients of variation
cTnT: Cardiac troponin
DPP: Diabetes Prevention Program
eGFR: Estimated glomerular filtration rate
FPG: Fasting plasma glucose
GDM: Gestational diabetes mellitus
HOMA2-S: Homeostasis model assessment2- sensitivity
hsCRP: high-sensitive-C-reactive protein
IADPSG: International Association of Diabetes and Pregnancy Study Group
IL-6: Interleukin-6
ISI: Insulin sensitivity index
IQR: Interquartile range
NP: Natriuretic peptide
NT-proBNP: N-terminal pro-B-type natriuretic peptide
OGTT: Oral glucose tolerance test
RAAS: Renin–angiotensin–aldosterone-system
WHO-IEC: World Health Organization-International Expert Committee

## References

[1] Volgman Annabelle S, Palaniappan Latha S, Aggarwal Neelum T, Gupta M, Khandelwal A, Krishnan Aruna V, et al. Atherosclerotic Cardiovascular Disease in South asians in the United States: epidemiology, risk factors, and treatments: A Scientific Statement from the American Heart Association. Circulation. 2018;138(1):e1–34. 29794080 10.1161/CIR.0000000000000580

[2] Dennison RA, Chen ES, Green ME, Legard C, Kotecha D, Farmer G, et al. The absolute and relative risk of type 2 diabetes after gestational diabetes: a systematic review and meta-analysis of 129 studies. Diabetes Res Clin Pract. 2021;171:108625. 33333204 10.1016/j.diabres.2020.108625 PMC7610694

[3] Farrar D, Fairley L, Santorelli G, Tuffnell D, Sheldon TA, Wright J, et al. Association between hyperglycaemia and adverse perinatal outcomes in south Asian and white British women: analysis of data from the born in Bradford cohort. Lancet Diabetes Endocrinol. 2015;3(10):795–804. 26355010 10.1016/S2213-8587(15)00255-7 PMC4673084

[4] Daly B, Toulis KA, Thomas N, Gokhale K, Martin J, Webber J, et al. Increased risk of ischemic heart disease, hypertension, and type 2 diabetes in women with previous gestational diabetes mellitus, a target group in general practice for preventive interventions: a population-based cohort study. PLoS Med. 2018;15(1):e1002488. 29337985 10.1371/journal.pmed.1002488 PMC5770032

[5] Kramer CK, Campbell S, Retnakaran R. Gestational diabetes and the risk of cardiovascular disease in women: a systematic review and meta-analysis. Diabetologia. 2019;62(6):905–14. 30843102 10.1007/s00125-019-4840-2

[6] Gunderson Erica P, Sun B, Catov Janet M, Carnethon M, Lewis Cora E, Allen Norrina B et al. Gestational diabetes history and glucose tolerance after pregnancy Associated with coronary artery calcium in women during midlife: the CARDIA Study. Circulation. 2021;0(0). 10.1161/CIRCULATIONAHA.120.047320 PMC7940578 33517667

[7] Gruden G, Landi A, Bruno G. Natriuretic peptides, Heart, and adipose tissue: New findings and Future Developments for Diabetes Research. Diabetes Care. 2014;37(11):2899–908. 25342830 10.2337/dc14-0669

[8] Malachias MVB, Wijkman MO, Bertoluci MC. NT-proBNP as a predictor of death and cardiovascular events in patients with type 2 diabetes. Diabetol Metab Syndr. 2022;14(1):64. 35501909 10.1186/s13098-022-00837-6 PMC9063067

[9] McCullough PA, Nowak RM, McCord J, Hollander JE, Herrmann HC, Steg PG, et al. B-Type natriuretic peptide and Clinical Judgment in Emergency diagnosis of heart failure. Circulation. 2002;106(4):416–22. 12135939 10.1161/01.CIR.0000025242.79963.4C

[10] Wang TJ, Larson MG, Levy D, Benjamin EJ, Leip EP, Omland T, et al. Plasma natriuretic peptide levels and the risk of Cardiovascular events and death. N Engl J Med. 2004;350(7):655–63. 14960742 10.1056/NEJMoa031994

[11] Pop-Busui R, Januzzi JL, Bruemmer D, Butalia S, Green JB, Horton WB, et al. Heart failure: an underappreciated complication of diabetes. A Consensus Report of the American Diabetes Association. Diabetes Care. 2022;45(7):1670–90. 35796765 10.2337/dci22-0014 PMC9726978

[12] American Diabetes Association Professional Practice C. 10. Cardiovascular Disease and Risk Management: Standards of Care in Diabetes—2024. Diabetes Care. 2023;47(Supplement_1):S179-S218. 10.2337/dc24-S010 PMC10725811 38078592

[13] Ndumele CE, Matsushita K, Sang Y, Lazo M, Agarwal SK, Nambi V, et al. N-Terminal Pro-brain Natriuretic peptide and heart failure risk among individuals with and without obesity. Circulation. 2016;133(7):631–8. 26746175 10.1161/CIRCULATIONAHA.115.017298 PMC4758863

[14] Lazo M, Young JH, Brancati FL, Coresh J, Whelton S, Ndumele CE, et al. NH2-Terminal pro–brain natriuretic peptide and risk of diabetes. Diabetes. 2013;62(9):3189–93. 23733199 10.2337/db13-0478 PMC3749338

[15] Pfister R, Sharp S, Luben R, Welsh P, Barroso I, Salomaa V, et al. Mendelian randomization study of B-type natriuretic peptide and type 2 diabetes: evidence of causal association from population studies. PLoS Med. 2011;8(10):e1001112–e. 22039354 10.1371/journal.pmed.1001112 PMC3201934

[16] Everett BM, Cook NR, Chasman DI, Magnone MC, Bobadilla M, Rifai N, et al. Prospective evaluation of B-type natriuretic peptide concentrations and the risk of type 2 diabetes in women. Clin Chem. 2013;59(3):557–65. 23288489 10.1373/clinchem.2012.194167 PMC3694412

[17] Ramos HR, Birkenfeld AL, de Bold AJ. INTERACTING DISCIPLINES: Cardiac natriuretic peptides and obesity: perspectives from an endocrinologist and a cardiologist. Endocr Connections. 2015;4(3):R25–36. 10.1530/EC-15-0018 PMC4485177 26115665

[18] Sattar N, Gill JM. Type 2 diabetes in migrant south asians: mechanisms, mitigation, and management. Lancet Diabetes Endocrinol. 2015;3(12):1004–16. 26489808 10.1016/S2213-8587(15)00326-5

[19] Chambers JC, Eda S, Bassett P, Karim Y, Thompson SG, Gallimore JR, et al. C-Reactive protein, insulin resistance, central obesity, and Coronary Heart Disease Risk in Indian asians from the United Kingdom compared with European whites. Circulation. 2001;104(2):145–50. 11447077 10.1161/01.CIR.104.2.145

[20] Sharma A, Lee-Ødegård S, Qvigstad E, Sommer C, Sattar N, Gill JMR, et al. β-Cell function, Hepatic Insulin Clearance, and insulin sensitivity in south Asian and nordic women after gestational diabetes Mellitus. Diabetes. 2022;71(12):2530–8. 36112815 10.2337/db22-0622

[21] Assay RDEN-p. 510(k) Substantial equivalence determination decision summary assay only template https://www.accessdata.fda.gov/cdrh_docs/reviews/K063662.pdf. Accessed 27 Jun 2024.

[22] Region WWP. The Asia-Pacific perspective: Redefining obesity. 2000. https://iris.who.int/handle/10665/206936. Accessed 27 Jan 2022.

[23] Misra A. Ethnic-specific criteria for classification of body Mass Index: a perspective for Asian indians and American Diabetes Association position Statement. Diabetes Technol Ther. 2015;17(9):667–71. 25902357 10.1089/dia.2015.0007 PMC4555479

[24] Wallace TM, Levy JC, Matthews DR. Use and abuse of HOMA modeling. Diabetes Care. 2004;27(6):1487. 15161807 10.2337/diacare.27.6.1487

[25] Diabetes Trials Unit: Oxford Centre for Diabetes Endocrinology and Metabolism. HOMA2 calculator [Internet]. 2019. https://www.dtu.ox.ac.uk/homacalculator/. Accessed 12 Mar 2022.

[26] Fujita Y, Kouda K, Ohara K, Nakamura H, Iki M. Leptin mediates the relationship between fat mass and blood pressure: the Hamamatsu School-based health study. Med (Baltim). 2019;98(12):e14934. 10.1097/MD.0000000000014934 PMC6708678 30896657

[27] Gupta DK, Claggett B, Wells Q, Cheng S, Li M, Maruthur N, et al. Racial differences in circulating natriuretic peptide levels: the atherosclerosis risk in communities Study. J Am Heart Assoc. 2015;4(5):e001831. 25999400 10.1161/JAHA.115.001831 PMC4599412

[28] Patel N, Russell GK, Musunuru K, Gutierrez OM, Halade G, Kain V, et al. Race, Natriuretic Peptides, and high-carbohydrate challenge: a clinical trial. Circ Res. 2019;125(11):957–68. 31588864 10.1161/CIRCRESAHA.119.315026 PMC7033629

[29] Khan AM, Cheng S, Magnusson M, Larson MG, Newton-Cheh C, McCabe EL, et al. Cardiac natriuretic peptides, obesity, and insulin resistance: evidence from two community-based studies. J Clin Endocrinol Metab. 2011;96(10):3242–9. 21849523 10.1210/jc.2011-1182 PMC3200240

[30] Walford GA, Ma Y, Christophi CA, Goldberg RB, Jarolim P, Horton E, et al. Circulating natriuretic peptide concentrations reflect changes in insulin sensitivity over time in the diabetes Prevention Program. Diabetologia. 2014;57(5):935–9. 24554005 10.1007/s00125-014-3183-2 PMC4158711

[31] Baldassarre S, Fragapani S, Panero A, Fedele D, Pinach S, Lucchiari M, et al. NTproBNP in insulin-resistance mediated conditions: overweight/obesity, metabolic syndrome and diabetes. The population-based Casale Monferrato Study. Cardiovasc Diabetol. 2017;16(1):119. 28946871 10.1186/s12933-017-0601-z PMC5613356

[32] Kim F, Biggs ML, Kizer JR, Brutsaert EF, de Filippi C, Newman AB, et al. Brain natriuretic peptide and insulin resistance in older adults. Diabet Med. 2017;34(2):235–8. 27101535 10.1111/dme.13139 PMC5074911

[33] Ciardullo S, Rea F, Cannistraci R, Muraca E, Perra S, Zerbini F, et al. NT-ProBNP and mortality across the spectrum of glucose tolerance in the general US population. Cardiovasc Diabetol. 2022;21(1):236. 36344968 10.1186/s12933-022-01671-w PMC9641859

[34] Sanchez OA, Duprez DA, Bahrami H, Daniels LB, Folsom AR, Lima JA, et al. The associations between metabolic variables and NT-proBNP are blunted at pathological ranges: the multi-ethnic study of atherosclerosis. Metabolism. 2014;63(4):475–83. 24388001 10.1016/j.metabol.2013.11.017 PMC3965618

[35] Peters MJL, Ghouri N, McKeigue P, Forouhi NG, Sattar N. Circulating IL-6 concentrations and associated anthropometric and metabolic parameters in south Asian men and women in comparison to European whites. Cytokine. 2013;61(1):29–32. 23026295 10.1016/j.cyto.2012.09.002

[36] Anand SS, Tarnopolsky MA, Rashid S, Schulze KM, Desai D, Mente A, et al. Adipocyte hypertrophy, fatty liver and metabolic risk factors in South asians: the Molecular Study of Health and Risk in ethnic groups (mol-SHARE). PLoS ONE. 2011;6(7):e22112. 21829446 10.1371/journal.pone.0022112 PMC3145635

[37] Dewan P, Jhund PS, Shen L, Petrie MC, Abraham WT, Atif Ali M, et al. Heart failure with reduced ejection fraction: comparison of patient characteristics and clinical outcomes within Asia and between Asia, Europe and the Americas. Eur J Heart Fail. 2019;21(5):577–87. 30536678 10.1002/ejhf.1347 PMC6607486

[38] Packer M. Leptin-Aldosterone-Neprilysin Axis. Circulation. 2018;137(15):1614–31. 29632154 10.1161/CIRCULATIONAHA.117.032474

[39] Sniderman AD, Bhopal R, Prabhakaran D, Sarrafzadegan N, Tchernof A. Why might South asians be so susceptible to central obesity and its atherogenic consequences? The adipose tissue overflow hypothesis. Int J Epidemiol. 2007;36(1):220–5. 17510078 10.1093/ije/dyl245

[40] Caleyachetty R, Barber TM, Mohammed NI, Cappuccio FP, Hardy R, Mathur R, et al. Ethnicity-specific BMI cutoffs for obesity based on type 2 diabetes risk in England: a population-based cohort study. Lancet Diabetes Endocrinol. 2021;9(7):419–26. 33989535 10.1016/S2213-8587(21)00088-7 PMC8208895

[41] Narayan KMV, Kanaya AM. Why are south asians prone to type 2 diabetes? A hypothesis based on underexplored pathways. Diabetologia. 2020;63(6):1103–9. 32236731 10.1007/s00125-020-05132-5 PMC7531132

[42] Echouffo-Tcheugui JB, Zhang S, McEvoy JW, Juraschek SP, Fang M, Ndumele CE, et al. Insulin resistance and N-Terminal Pro-B-Type natriuretic peptide among healthy adults. JAMA Cardiol. 2023;8(10):989–95. 37672260 10.1001/jamacardio.2023.2758 PMC10483384

[43] Anjan VY, Loftus TM, Burke MA, Akhter N, Fonarow GC, Gheorghiade M, et al. Prevalence, clinical phenotype, and outcomes associated with normal B-type natriuretic peptide levels in heart failure with preserved ejection fraction. Am J Cardiol. 2012;110(6):870–6. 22681864 10.1016/j.amjcard.2012.05.014 PMC3432159

[44] Park JJ, Choi D-J, Yoon C-H, Oh I-Y, Jeon E-S, Kim J-J, et al. Prognostic value of C-Reactive protein as an inflammatory and N-Terminal Probrain Natriuretic peptide as a neurohumoral marker in Acute Heart failure (from the Korean Heart failure Registry). Am J Cardiol. 2014;113(3):511–7. 24315115 10.1016/j.amjcard.2013.10.022

[45] Dessì-Fulgheri P, Sarzani R, Tamburrini P, Moraca A, Espinosa E, Cola G et al. Plasma atrial natriuretic peptide and natriuretic peptide receptor gene expression in adipose tissue of normotensive and hypertensive obese patients. J Hypertens. 1997;15(12). 10.1097/00004872-199715120-00074 9488224

[46] Palmer SC, Yandle TG, Nicholls MG, Frampton CM, Richards AM. Regional clearance of amino-terminal pro-brain natriuretic peptide from human plasma. Eur J Heart Fail. 2009;11(9):832–9. 19605456 10.1093/eurjhf/hfp099

[47] Patel AP, Wang M, Kartoun U, Ng K, Khera AV. Quantifying and understanding the higher risk of atherosclerotic Cardiovascular Disease among south Asian individuals. Circulation. 2021;144(6):410–22. 34247495 10.1161/CIRCULATIONAHA.120.052430 PMC8355171

[48] Vistisen D, Witte DR, Brunner EJ, Kivimäki M, Tabák A, Jørgensen ME, et al. Risk of cardiovascular disease and death in individuals with prediabetes defined by different criteria: the Whitehall II study. Diabetes Care. 2018;41(4):899. 29453200 10.2337/dc17-2530 PMC6463620

