# Supplementary material for: N-terminal pro-B-type natriuretic peptide levels vary by ethnicity and are associated with insulin sensitivity after gestational diabetes mellitus
Source: Cardiovasc Diabetol. 2024 Aug 3;23:284. doi: 10.1186/s12933-024-02349-1 (PMC11298077; doi:10.1186/s12933-024-02349-1)
Supplement: Supplementary file 1 — Supplementary Material 1 [file 12933_2024_2349_MOESM1_ESM.docx]

**Additional File 1:**

**Table S1.** Ethnic differences in cardiac, inflammation, adipokine, and insulin sensitivity markers by glucose tolerance status

| **Different** |  | **Normal glucose tolerance** | | **Prediabetes/diabetes** | |
| --- | --- | --- | --- | --- | --- |
| **Markers** |  | South Asian | Nordic | South Asian | Nordic |
|  |  | n=48 [30%] | n=57 [53%] | n=114 [70%] | n=50 [47%] |
| Cardiac | NT-proBNP | 29 (17-38) | 40 (22-58)* | 22 (14-40) | 44 (21-74)*** |
|  | Troponin T | 1.5 (1.5-1.5) | 1.5 (1.5-1.5) | 1.5 (1.5-1.5) | 1.5 (1.5-1.5) |
| Inflammation | CRP | 1.7 (0.8-3.2) | 0.8 (0.3-2.3)** | 2.7 (1.3-4.6) | 2.2 (0.9-5.7) |
|  | Interleukin 6 | 2.2 (1.5-3.2) | 1.5 (1.5-2.1)*** | 2.3 (1.5-3.5) | 1.9 (1.5-3.1) |
| Adipokines | Leptin | 1620 (1136-2198) | 1036 (573-1430)** | 1669 (1199-2602) | 1824 (1038-2906) |
|  | Adiponectin | 7.4 (5.6-9.5) | 10.9 (8.2-13.5)*** | 6.8 (5.1-9.4) | 8.7 (6.3-13.6)** |
| Insulin sensitivity | HOMA2-S | 61 (44-87) | 102 (70-138)*** | 44 (32-69) | 63 (46-87)** |
|  | Matsuda-ISI | 3.1 (2.4-4.2) | 5.6 (3.5-7.2)*** | 2.2 (1.6-3.3) | 3.2 (2.1-4.2)*** |

Data presented as median (IQR) or number [n].

HOMA2-S: HOMA2-sensitivity, ISI: insulin sensitivity index, NT-pro BNP: N-terminal pro B-type natriuretic peptide

**p* ≤ 0.05, ***p* ≤ 0.01, ****p* ≤ 0.001 for South Asian vs. Nordic women

**Table S2**. Correlation between NT-proBNP and metabolic markers in South Asian and Nordic women

|  |  | | **South Asian** | **Nordic** | **All** |
| --- | --- | --- | --- | --- | --- |
|  | |  | n=162 [31%] | n=107 [53%] | n=269 |
| BMI (kg/m^2)^ | |  | -0.086 (0.274) | -0.006 (0.954) | **-**0.065 (0.287) |
| Waist-to-height ratio | |  | -0.111 (0.163) | -0.096 (0.325) | -0.153 (0.012) |
| Systolic blood pressure (mmHg) | |  | 0.037 (0.641) | 0.130 (0.181) | 0.093 (0.128) (0 |
| eGFR (mL/min/1.73m^2^) | |  | -0.200 (0.011) | -0.050 (0.611) | **-0.206 (<0.001)** |
| Fasting plasma glucose (mmol/L) | |  | -0.068 (0.390) | -0.092 (0.345) | -0.083 (0.177) |
| 2-h OGTT glucose (mmol/L) | |  | -0.081 (0.306) | 0.057 (0.561) | -0.071 (0.247) |
| HbA_1c_ (mmol/mol) | |  | -0.108 (0.171) | -0.083 (0.397) | **-**0.169 (0.005) |
| Fasting insulin (pmol/L) | |  | -0.255 (0.001) | -0.288 (0.003) | **-0.361 (<0.001)** |
| Fasting c-peptide (pmol/L) | |  | -0.202 (0.011) | -0.254 (0.008) | **-0.304 (<0.001)** |
| C-reactive protein (mg/L) | |  | -0.047 (0.554) | 0.079 (0.416) | -0.061 (0.316) |
| Interleukin 6^‡^ (pg/ml) | |  | -0.113 (0.153) | -0.134 (0.170) | -0.194 (0.001) |
| Leptin (pmol/L) | |  | -0.156 (0.065) | -0.043 (0.666) | -0.146 (0.022) |
| Adiponectin (mg/L) | |  | 0.232 (0.006) | 0.168 (0.087) | **0.293 (<0.001)** |
| HOMA2-S | |  | 0.255 (0.001) | 0.280 (0.004) | **0.355 (<0.001)** |
| Matsuda ISI | |  | **0.291 (<0.001)** | 0.243 (0.013) | **0.369 (<0.001)** |

Data presented as r-spearman correlation (p-value).

HOMA-S: HOMA2-sensitivity, Matsuda ISI: Matsuda insulin sensitivity index, NT-proBNP: N-terminal pro B-type natriuretic peptide

For all analyses p-values < 0.001 were considered significant to reduce the number of sporadic findings due to multiple testing.

| **Table S3.** Predictors of NT-proBNP (ng/L) concentrations (log transformed) in unadjusted and adjusted linear regression analyses | | | | | | | | | | | | |
| --- | --- | --- | --- | --- | --- | --- | --- | --- | --- | --- | --- | --- |
|  | **Unadjusted** | | **Model 1** | | **Model 2** | | **Model 3** | | **Model 4** | | **Model 5** | |
|  | **Crude B** | ***P*** | **Std B** | ***p*** | **Std B** | ***p*** | **Std B** | ***p*** | **Std B** | ***p*** | **Std B** | ***P*** |
| **Ethnicity** | -0.201 | <0.001 | -0.155 | 0.015 | -0.161 | 0.011 | -0.133 | 0.037 | -0.066 | 0.333 | 0.029 | 0.701 |
| **Age (years)** | 0.116 | 0.057 | 0.032 | 0.632 | 0.041 | 0.532 | 0.048 | 0.473 | 0.052 | 0.435 | 0.032 | 0.636 |
| **Systolic blood pressure (mmHg)** | 0.105 | 0.085 | 0.065 | 0.286 | 0.093 | 0.124 | 0.110 | 0.079 | 0.095 | 0.125 | 0.098 | 0.129 |
| **eGFR (mL/min/1.73m^2^)** | -0.171 | 0.005 | -0.117 | 0.084 | -0.116 | 0.085 | -0.122 | 0.071 | -0.106 | 0.115 | -0.097 | 0.156 |
| **Interleukin-6 (pg/mL)** | -0.064 | 0.294 |  |  | -0.022 | 0.711 | -0.042 | 0.525 | -0.023 | 0.732 | -0.019 | 0.774 |
| **Body mass index** | -0.012 | 0.844 |  |  |  |  | -0.059 | 0.386 | 0.031 | 0.655 | 0.144 | 0.057 |
| **Adiponectin (mg/L)** | 0.238 | <0.001 |  |  |  |  |  |  | 0.197 | 0.003 | 0.145 | 0.036 |
| **Matsuda ISI** | 0.265 | <0.001 |  |  |  |  |  |  |  |  | 0.264 | 0.001 |

Model 1 (ethnicity, age, systolic blood pressure, eGFR), model 2 (model 1 + interleukin -6), model 3 (model 2 + body mass index), model 4 (model 3 + adiponectin) and model 5 (model 4 + Matsuda ISI (insulin sensitivity index)).

For all analyses p-values <0.05 were considered significant

**Table S4.** Predictors of NT-proBNP (ng/L) concentrations (log transformed) in South Asian and Nordic women

| **South Asian** | **logNT-proBNP** | | |
| --- | --- | --- | --- |
|  | **Standardized B** | ***p*** | **95% C.I.** |
| Age (years) | 0.145 | 0.114 | -0.003 to 0.031 |
| Systolic blood pressure (mmHg) | 0.061 | 0.469 | -0.005 to 0.010 |
| GFR (mL/min/1.73m^2^) | -0.135 | 0.141 | -0.013 to 0.002 |
| Interleukin-6 (pg/mL) | 0.185 | **0.047** | 0.001 to 0.096 |
| Leptin (pmol/L) | -0.255 | **0.009** | 0.000 to 0.000 |
| Adiponectin (mg/L) | 0.103 | 0.241 | -0.009 to 0.034 |
| Matsuda ISI | 0.214 | **0.030** | 0.005 to 0.106 |

| **Nordic** | **logNT-proBNP** | | |
| --- | --- | --- | --- |
|  | **Standardized B** | ***p*** | **95% C.I.** |
| Age (years) | -0.099 | 0.353 | -0.025 to 0.009 |
| Systolic blood pressure (mmHg) | 0.144 | 0.184 | -0.002 to 0.013 |
| GFR (mL/min/1.73m^2^) | -0.068 | 0.526 | -0.010 to 0.005 |
| Interleukin-6 (pg/mL) | -0.041 | 0.687 | -0.061 to 0.040 |
| Leptin (pmol/L) | -0.076 | 0.514 | 0.000 to 0.000 |
| Adiponectin (mg/L) | 0.116 | 0.318 | -0.009 to 0.027 |
| Matsuda ISI | 0.277 | **0.020** | 0.007 to 0.080 |

Linear regression analyses adjusted for ethnicity, age, BMI, systolic blood pressure, eGFR, interleukin-6, leptin, adiponectin and Matsuda ISI (Model 5).

ISI: insulin sensitivity index, NT-pro BNP: N-terminal pro B-type natriuretic peptide

For all analyses p-values <0.05 were considered significant.

**Table S5.** Predictors of NT-proBNP (ng/L) concentrations (log transformed) in women with normal glucose tolerance and prediabetes/diabetes

| **Normal glucose toleranse** | **logNT-proBNP** | | |
| --- | --- | --- | --- |
|  | **Standardized B** | ***p*** | **95% C.I.** |
| Ethnicity | -0.129 | 0.315 | -0.294 to 0.096 |
| Age (years) | -0.252 | **0.038** | -0.039 to -0.001 |
| Systolic blood pressure (mmHg) | 0.149 | 0.172 | -0.003 to 0.015 |
| GFR (mL/min/1.73m^2^) | -0.150 | 0.264 | -0.016 to 0.004 |
| Interleukin-6 (pg/mL) | 0.103 | 0.339 | -0.028 to 0.081 |
| Leptin (pmol/L) | -0.268 | **0.030** | 0.000 to 0.000 |
| Adiponectin (mg/L) | -0.115 | 0.369 | -0.027 to 0.010 |
| Matsuda ISI | 0.093 | 0.480 | -0.024 to 0.050 |
|  |  |  |  |
| **Prediabetes or diabetes** | **logNT-proBNP** | | |
|  | **Standardized B** | ***p*** | **95% C.I.** |
| Ethnicity | -0.002 | 0.980 | -0.158 to 0.154 |
| Age (years) | 0.141 | 0.091 | -0.002 to 0.030 |
| Systolic blood pressure (mmHg) | 0.118 | 0.152 | -0.002 to 0.011 |
| GFR (mL/min/1.73m^2^) | -0.138 | 0.103 | -0.011 to 0.001 |
| Interleukin-6 (pg/mL) | 0.036 | 0.666 | -0.034 to 0.053 |
| Leptin (pmol/L) | 0.001 | 0.987 | 0.000 to 0.000 |
| Adiponectin (mg/L) | 0.167 | 0.057 | -0.001 to 0.038 |
| Matsuda ISI | 0.324 | **<0.001** | 0.036 to 0.138 |

Linear regression analyses adjusted for ethnicity, age, BMI, systolic blood pressure, eGFR, interleukin-6, leptin, adiponectin and Matsuda ISI (Model 5).

ISI: insulin sensitivity index, NT-pro BNP: N-terminal pro B-type natriuretic peptide

For all analyses p-values <0.05 were considered significant.

**Table S6.** Mediation analyses of ethnic differences in logNT-proBNP

| **Ethnicity** | **Beta** | **Beta change (%)** | **Odds Ratio** | **p-value** | **95% C.I.** |
| --- | --- | --- | --- | --- | --- |
| **LogNT-proBNP** | -1.091 | - | 0.336 | 0.001 | 0.173 to 0.654 |
| **+ Age** | -1.038 | 4.9 | 0.354 | 0.003 | 0.179 to 0.703 |
| **+ BMI** | -1.091 | 0.0 | 0.336 | 0.001 | 0.173 to 0.654 |
| **+ Systolic blood pressure** | -1.047 | 4.0 | 0.351 | 0.002 | 0.179 to 0.687 |
| **+eGFR** | -0.928 | 14.9 | 0.395 | 0.008 | 0.199 to 0.786 |
| **+ IL-6** | -1.098 | 0.6 | 0.334 | 0.001 | 0.170 to 0.655 |
| **+ Leptin** | -1.104 | 1.2 | 0.331 | 0.002 | 0.165 to 0.667 |
| **+ Adiponectin** | -0.648 | 40.6 | 0.523 | 0.076 | 0.256 to 1.070 |
| **+ Matsuda ISI** | -0.515 | 52.8 | 0.598 | 0.159 | 0.292 to 1.223 |
|  |  |  |  |  |  |

Mediation analyses of ethnic differences in logNT-proBNP, adjusted separately for age (years), BMI (kg/m^2^), systolic blood pressure (mmHg), eGFR (ml/min/1.73m^2^), interleukin-6 (pg/mL), leptin (pmol/L), adiponectin (mg/L) and insulin sensitivity (by Matsuda insulin sensitivity index, ISI). Insulin sensitivity, adiponectin and eGFR mediated 56.4%, 40.6% and 14.9% of the ethnic difference in logNT-proBNP, respectively. Data are log β-coefficients for logNT-proBNP with 95% CI.

**Fig. S1.** NT-proBNP distribution across ethnicities


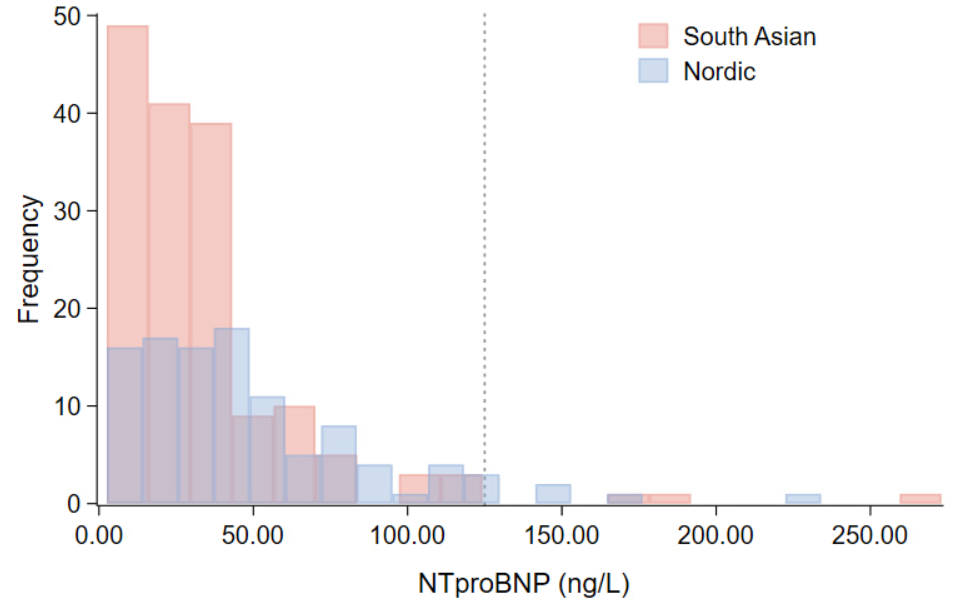


**Supplementary Fig. 1.** NT-proBNP (ng/L) distribution for South Asian (red) and Nordic (blue) women. In total, 1.9% (n = 3) of the South Asian and 5.6% (n =6) of the Nordic women had NT-proBNP levels ≥ 125 ng/L (vertical dotted line).
