# Supplementary figures and images for: N-terminal pro-B-type natriuretic peptide levels vary by ethnicity and are associated with insulin sensitivity after gestational diabetes mellitus
Source: Cardiovasc Diabetol. 2024 Aug 3;23:284. doi: 10.1186/s12933-024-02349-1 (PMC11298077; doi:10.1186/s12933-024-02349-1)

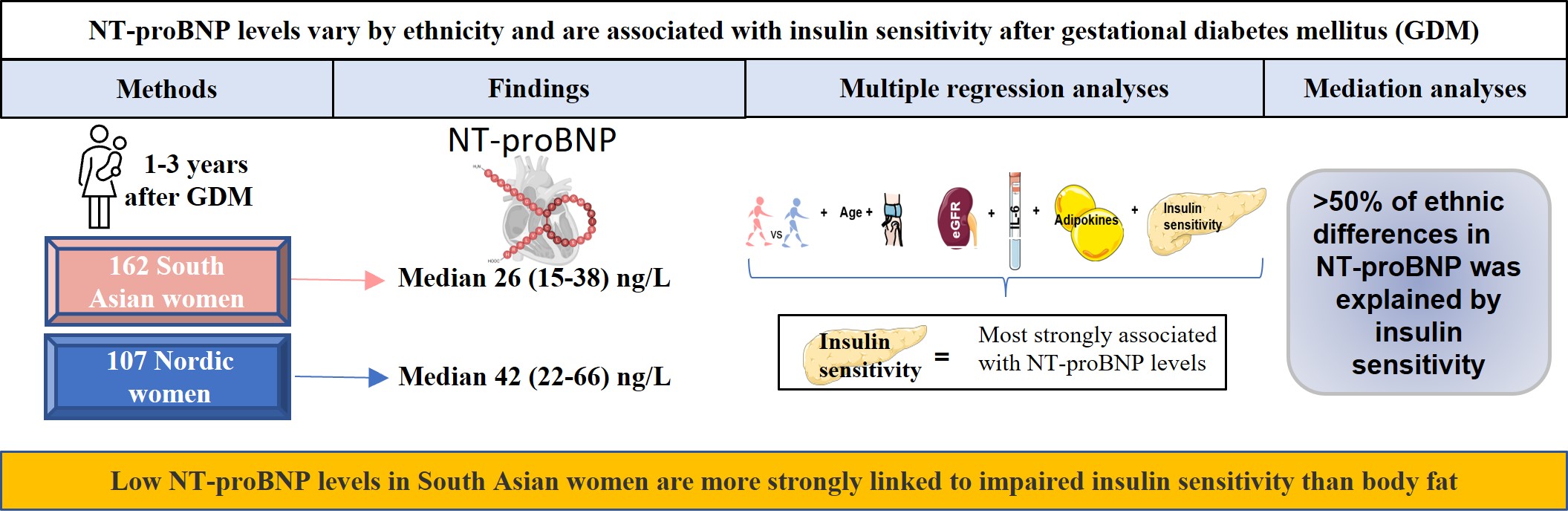

Supplement: Supplementary file 2 — Supplementary Material 2 [file 12933_2024_2349_MOESM2_ESM.jpg]
